# Thermomechanical Responses of Cryogenic Composite Pressure Vessels with Different Dome Aspect Ratios

**DOI:** 10.3390/ma19194113

**Published:** 2026-09-26

**Authors:** Wenbin Gui, Zengshi Xu, Qingchang Guo, Liran Ma, Jianbin Luo

**Affiliations:** 1 State Key Laboratory of Tribology, Tsinghua University, Beijing 100084, China; 2 Wuhan Institute of Marine Electric Propulsion, Wuhan 430064, China

**Keywords:** cryogenic pressure vessel, dome aspect ratio, composite overwrap, thermomechanical response

## Abstract

Large-scale metal-lined composite overwrapped pressure vessels are promising for cryogenic liquid oxygen storage, but their response is governed by the interaction among dome geometry, thermal mismatch and internal pressure. This study investigates the influence of dome aspect ratio (DAR), defined as the ratio of the major to minor semi-axis of an ellipsoidal dome, on vessel thermomechanical behavior. Six DAR values from 1.25 to 2.50 were analyzed using a three-dimensional finite element model with temperature-dependent properties of Type 304 stainless steel and T700-grade carbon/epoxy. Three loading conditions were considered including 1.0 MPa internal pressure at 293 K, cooling from 293 to 90 K, and pressurization at 90 K. At 293 K, increasing DAR intensified stress localization near the dome–cylinder transition. Cryogenic cooling produced incompatible free deformations between the liner and overwrap, generating substantial constrained thermal stresses after bonding. Subsequent pressurization further increased the liner stress but partly offset the cooling-induced compressive stress in the overwrap. Under cryogenic and combined loading, DAR had little influence on the central cylindrical section, but markedly changed the stress magnitude, sign and localization in the domes and transition regions. These findings indicate that dome shapes for cryogenic vessels should not be selected solely from room-temperature pressure-based conclusions, because thermal mismatch and cryogenic pressurization can fundamentally alter the structural response.

## 1. Introduction

The development of lightweight and reliable cryogenic storage vessels has become increasingly important for advanced energy and propulsion systems in which cryogenic fluids such as liquid oxygen are used as high-density working media [1,2]. Compared with conventional gaseous storage, cryogenic storage can provide a much higher storage density, but it also imposes more stringent requirements on the structural integrity, sealing performance and low-temperature adaptability of the vessel [3]. These requirements become more critical for large-scale storage structures, where the increase in vessel diameter and overall dimensions may lead to higher structural weight, more complex deformation behavior and greater sensitivity to local stress concentrations [4]. Metal-lined composite overwrapped pressure vessels (COPVs) provide an effective structural concept for this application [5]. In such vessels, the metallic liner ensures gas-tightness, containment of cryogenic fluids such as liquid oxygen, and dimensional stability, while the composite overwrap carries the major portion of the mechanical load with high specific strength and stiffness [6]. Therefore, large-scale metal-lined fully wrapped composite vessels are considered a promising solution for lightweight cryogenic storage of fluids such as liquid oxygen, provided that their structural response under cryogenic service conditions can be accurately understood and properly controlled [7].

However, the structural behavior of metal-lined composite vessels becomes considerably more complicated when the vessel is cooled from ambient temperature to the liquid oxygen temperature range [8]. The metallic liner, carbon fibers and polymer matrix exhibit different thermal contraction characteristics and temperature-dependent mechanical responses [9]. As a result, cooling alone can generate a non-negligible initial stress field before the application of internal pressure. This effect is not limited to the macroscopic mismatch between the metallic liner and the composite overwrap; it also exists within the composite layer itself because the fiber and matrix phases contract differently under cryogenic conditions [10]. Previous studies have shown that such coefficient-of-thermal-expansion mismatch may lead to residual thermal stresses, matrix microcracking, interfacial stress concentration and changes in damage initiation behavior [8]. Therefore, the load-bearing response of cryogenic composite pressure vessels should be regarded as a thermo-mechanical coupling problem rather than a simple internal-pressure problem [11]. For large-scale vessels, this issue may become more pronounced because the accumulated thermal shrinkage displacement increases with structural dimension, and the deformation compatibility among the liner, composite overwrap, cylindrical section and dome region becomes more difficult to maintain. Consequently, the liner–composite interface and the dome–cylinder transition region may become particularly sensitive under cryogenic loading, where thermal contraction, geometric constraint and subsequent internal pressure jointly determine the actual stress state of the vessel [12,13].

The dome region is a design-sensitive part of a composite overwrapped pressure vessel because it connects the cylindrical section to the polar opening while exhibiting continuous changes in curvature, fiber orientation and local laminate thickness [14,15]. Unlike the cylindrical section, where winding angle and thickness are relatively uniform, dome geometry governs fiber-path evolution along the meridian and load transfer from the cylinder to the dome [16]. Previous studies show that dome contours strongly affect the stress state near the dome–cylinder transition, where curvature discontinuity, thickness variation and deformation incompatibility may introduce bending and interlaminar shear stresses not captured by simple netting-based methods [17]. From manufacturing and structural perspectives, geodesic and non-geodesic winding studies also indicate that fiber angle, tow-path stability, bandwidth, thickness accumulation and local reinforcement are closely coupled with dome geometry [18]. For ellipsoidal domes, the dome aspect ratio (DAR), defined as the ratio of the major radius to the minor radius, changes the meridional curvature, dome height and transition geometry, thereby affecting geodesic fiber-path distribution, stress redistribution in the liner and overwrap, and vessel load-bearing efficiency. DAR is therefore not merely a geometric parameter, but a key variable linking dome curvature, winding architecture, stress concentration and pressure-bearing performance [19].

Although dome geometry has been widely studied, most existing works still focus on pressure-dominated design. Dome contour, curvature, winding-angle evolution and local reinforcement affect stress distribution, burst pressure, failure location, structural efficiency and weight performance [20]. These studies provide useful guidance for hydrogen storage vessels and filament-wound COPVs, but mainly address internal-pressure response, material usage and manufacturability [21,22]. Research on composite cryogenic tanks has also highlighted challenges such as liquid oxygen compatibility, cryogenic mechanical degradation, microcracking and leakage resistance [23,24]. However, these studies emphasize materials, leakage prevention, damage mechanisms and manufacturing feasibility, while the influence of structural geometry on cryogenic thermo-mechanical response remains insufficiently clarified. For large-scale metal-lined liquid oxygen vessels, DAR may affect geodesic winding architecture, liner–composite deformation compatibility and cooling-induced thermal-stress redistribution. Conventional dome-design conclusions derived from room-temperature or pressure-dominated COPVs therefore cannot be directly extended to large cryogenic vessels. The interaction between DAR, thermal mismatch and internal pressure requires systematic investigation to support lightweight and reliable cryogenic COPV design.

Based on these considerations, this study investigates the influence of dome aspect ratio (DAR) on the thermomechanical response of a large-scale metal-lined composite overwrapped pressure vessel for cryogenic liquid oxygen storage. Six ellipsoidal dome configurations with DAR values ranging from 1.25 to 2.50 are established using a three-dimensional finite element model incorporating the temperature-dependent properties of Type 304 stainless steel and T700-grade carbon/epoxy. Three loading conditions are considered: 1 MPa internal pressure at 293 K, uniform cooling from 293 to 90 K without internal pressure, and pressurization at 90 K. Particular attention is given to the deformation mismatch between the liner and composite overwrap, the constrained thermal stresses generated after bonding, and the redistribution of stress in the cylindrical, dome, and transition regions. The results clarify how dome geometry alters the vessel response under room-temperature, cryogenic, and combined loading, and provide a basis for assessing the applicability of conventional room-temperature dome-design conclusions to large-scale cryogenic metal-lined composite pressure vessels.

## 2. Numerical Model and Analysis Setup

The investigated structure is a large-scale metal-lined composite overwrapped pressure vessel designed for cryogenic liquid oxygen storage. The vessel consists of a cylindrical section, two ellipsoidal domes, and polar boss transition regions, with its longitudinal axis aligned with the global Y direction. The outer diameter and axial length of the cylindrical liner section are 2200 and 2580 mm, respectively, and the uniform liner thickness is 5 mm. DAR, calculated as the ratio of the major radius to the minor radius of the ellipsoidal dome, is selected as the primary geometric variable and assigned values of 1.25, 1.50, 1.75, 2.00, 2.25, and 2.50. A fillet radius of 55 mm is introduced at the boss–dome transition to reduce local geometric discontinuity and stress concentration. The overall vessel configuration and the definition of DAR are illustrated in Figure 1.

The metallic liner is made of Type 304 stainless steel and is represented using a temperature-dependent linear-elastic model in Abaqus. Young’s modulus is specified as 199.5, 207.7, and 214.1 GPa at 293, 193, and 90 K, respectively, based on published cryogenic material data [25], while the Poisson’s ratio is maintained at 0.27. The thermal-expansion coefficients of the liner and composite are treated as representative effective values over the investigated cooling interval, retaining the principal thermal-contraction mismatch between the stainless-steel liner and the anisotropic composite overwrap. The same material definitions are applied to all DAR configurations; accordingly, the calculated liner stresses are used primarily for comparing DAR-dependent stress redistribution and localization rather than for predicting post-yield plastic evolution. The composite overwrap is modelled in WoundSim as a T700-grade carbon fiber/epoxy material using orthotropic engineering constants. The elastic constants at 293 K are adopted from Ref. [12], whereas the transverse and shear moduli at 193 and 90 K are estimated according to the cryogenic stiffness trends reported in Ref. [26]. The longitudinal modulus and Poisson’s ratios are assumed temperature-independent because their variations are relatively limited, while the thermal expansion coefficients are taken from low-temperature measurements [27]. The resulting material properties are summarized in Table 1 and Table 2.

The finite element models are constructed and analyzed using Abaqus/CAE 2025, while the composite overwrap is generated using WoundSim 2026-2, with geodesic winding adopted for the helical plies and hoop winding applied over the cylindrical section. As illustrated in Figure 2, the layup consists of 32 plies with an individual thickness of 0.25 mm, giving a nominal composite thickness of 8 mm over the cylindrical section. The stacking sequence comprises eight geodesic helical plies, followed by eight hoop plies, another eight geodesic helical plies, and eight outer hoop plies. Here, G denotes a geodesic helical winding ply, whereas 90° denotes a hoop winding ply. For finite element implementation, each group of eight consecutive plies is represented by one equivalent layer with a nominal cylindrical thickness of 2 mm, resulting in the equivalent sequence [G/90°/G/90°]. This treatment preserves the stacking order, total cylindrical thickness, and helical-to-hoop thickness ratio, while WoundSim accounts for the variation in winding angle and helical-layer thickness over the dome region.

The metallic liner is discretised using C3D8R solid elements with an approximate global seed size of 50 mm. The composite overwrap is meshed independently using the WoundSim automatic meshing procedure, with separate circumferential and meridional mesh controls and two elements assigned through the thickness of each equivalent composite layer. To reduce computational cost while retaining the three-dimensional stress distribution, a 90° quarter model is adopted. Symmetry constraints are applied to the two radial cut planes. To eliminate the remaining rigid-body translation, an additional minimum constraint is applied to a small local node set near one polar end, for which only the axial displacement *U*_2_ in the global *Y* direction is fixed, while the transverse displacement components remain free. The liner and composite overwrap are connected using a tie constraint, representing an ideal bonded condition without interfacial sliding or separation. In the subsequent discussion, stresses reported near this bonded surface refer to stresses in the adjacent solid elements rather than interface tractions or debonding criteria. Three loading cases are considered: uniform cooling from room temperature (293 K) to 90 K without internal pressure, pressurization to 1 MPa at 293 K, and uniform cooling from 293 to 90 K followed by pressurization to 1 MPa. The cooling process is represented by a spatially uniform temperature field, while transient heat transfer and temperature gradients are neglected. The finite element model, boundary conditions, and loading cases are shown in Figure 3.

The present finite element model is primarily intended for a controlled comparison of DAR-dependent thermomechanical responses. The material properties are based on published cryogenic experimental data, while identical material definitions, winding architecture, interface treatment, boundary conditions, meshing strategy, and loading sequence are maintained for all DAR configurations. Therefore, the numerical results are mainly interpreted in terms of relative deformation and stress-redistribution trends. Dedicated experimental validation, including reduced-scale cryogenic testing, will be considered in future work to further assess the quantitative predictive capability of the model.

## 3. Results and Discussion

### 3.1. Effect of Dome Aspect Ratio Under Room-Temperature Pressurization

To establish the baseline structural response before introducing cryogenic effects, the influence of DAR under room-temperature (RT) internal pressure is first examined. Figure 4 presents the fiber-direction lamina stress distributions of the composite overwrap for the six dome aspect ratios. Although alternating longitudinal stress bands remain visible over the cylindrical section for all configurations, the stress state changes markedly with DAR, particularly in the dome and dome–cylinder transition regions. At DAR = 1.25, the fiber-direction stress is distributed relatively broadly over the cylindrical section and both domes. As DAR increases, the dome becomes shallower, and the stress distribution gradually shifts toward the dome shoulder and the dome–cylinder junction. The maximum tensile stress increases from approximately 73.4 MPa at DAR = 1.25 to 299.2 MPa at DAR = 2.50, while increasingly pronounced compressive zones also develop near the transition regions. This indicates that increasing DAR changes the load-transfer path rather than uniformly increasing the stress throughout the overwrap. Smaller DAR values provide a longer and smoother meridional transition, whereas larger DAR values compress the load-transfer region and intensify the local tensile–compressive stress gradient [20], particularly when DAR exceeds approximately 2.00.

To further clarify the origin of this redistribution, Figure 5 isolates the von Mises stress in the metallic liner. For DAR = 1.25 and 1.50, the cylindrical section constitutes the principal high-stress region, while the stresses over most of the dome surface remain lower. The stress distribution is comparatively uniform along the cylinder, and no sharply localized peak is observed at the dome shoulder. When DAR increases to 1.75, elevated stress zones begin to emerge near the dome–cylinder junction. This tendency becomes dominant for DAR ≥ 2.00. The maximum liner stress increases from approximately 219.9 MPa at DAR = 2.00 to 288.4 MPa at DAR = 2.25 and 355.7 MPa at DAR = 2.50, while the highest-stress region contracts into narrow circumferential bands adjacent to the upper and lower dome–cylinder transitions. At the same time, the stress level over the central cylindrical section becomes less dominant, demonstrating that the increase in the maximum stress is caused primarily by localization rather than by a uniform increase throughout the liner.

This transition can be attributed to the coupled effects of dome geometry, winding architecture, and deformation compatibility between the liner and composite overwrap, as illustrated in Figure 6. Under internal pressure, the cylindrical section tends to expand predominantly in the hoop direction, whereas the dome carries the load through coupled meridional and circumferential membrane action [28]. Increasing DAR reduces the dome height and produces a more abrupt change in curvature near the dome–cylinder junction. At the same time, the local geodesic winding orientation and thickness vary with the dome profile, while the hoop layers terminate near the transition region. Consequently, the radial and meridional deformation of the dome becomes less compatible with the nearly uniform expansion of the cylinder, introducing additional local bending and multiaxial stress into the liner [15]. The perfectly bonded composite overwrap further constrains this deformation through its anisotropic stiffness and spatially varying geodesic fiber orientation. The observed stress-concentration bands therefore reflect the combined effects of geometric transition and nonuniform winding-related restraint rather than curvature alone.

### 3.2. Cryogenic Deformation Mismatch and Stress Response

After establishing the room-temperature pressure response, the isolated effect of cryogenic cooling was examined using the vessel with DAR = 1.25. Figure 7 presents the stress distributions after uniform cooling from 293 to 90 K without internal pressure. The liner develops a self-equilibrated thermal stress field, with a maximum von Mises stress of approximately 412.5 MPa. Elevated stresses are mainly distributed around the dome–cylinder transition regions, while the central cylindrical section remains at a relatively lower level. The composite overwrap exhibits a considerably wider fiber-direction stress range, from approximately −438.6 to 471.7 MPa, and the four layer groups show clearly different responses. The helical layers extending over the complete vessel are more sensitive to the dome and transition geometry, whereas the hoop layers confined to the cylindrical section exhibit regularly alternating axial bands. These bands correspond to a periodic variation along the circumferential direction rather than a gradual variation along the vessel axis. Since a uniform temperature field was applied, this modulation is not caused by a thermal gradient, but is associated with the nonuniform restraint produced by the winding architecture and the compatibility between different layer groups.

To distinguish the intrinsic thermal deformation tendencies of the two components, the liner and composite overwrap were first cooled independently, as shown in Figure 8, with the displayed deformation magnified by a factor of 50. The metallic liner undergoes smooth contraction in both the circumferential and axial directions, with a maximum displacement of approximately 12.67 mm, while retaining a nearly axisymmetric profile. In contrast, the freely cooled composite overwrap exhibits a markedly different deformation mode, with a maximum displacement of approximately 9.97 mm. Its cylindrical section contracts circumferentially but extends in the axial direction, accompanied by pronounced curvature changes in the domes and near the dome–cylinder transitions.

The different deformation modes are governed by the thermal contraction characteristics of the two materials. The stainless-steel liner is nearly isotropic, so cooling produces contraction in both the axial and circumferential directions. The composite overwrap behaves differently because its thermal expansion coefficient is much smaller along the fibers than transverse to them [29]. In the cylindrical section, as illustrated in Figure 9, the geodesic fibers are oriented mainly along the vessel axis and therefore strongly restrain axial shortening, whereas the larger transverse contraction allows the cylinder diameter to decrease. As the circumference decreases, the circumferential projection of each helical fiber path becomes shorter. Since the fiber-direction thermal strain is small and the total length of the helical path changes only slightly, its axial projection must increase to maintain geometric compatibility, which appears macroscopically as axial elongation of the cylindrical section. The hoop layers oppose the reduction in diameter but cannot completely suppress it. Over the dome, the continuously varying geodesic angle and the termination of the hoop layers near the dome–cylinder junction make the thermal contraction less uniform, producing the more pronounced curvature changes observed in these regions [30].

Figure 10a compares the independently cooled liner and composite overwrap by superposing their free-deformation profiles. The two components do not contract toward the same shape. The liner exhibits a relatively uniform reduction in both diameter and axial length, whereas the composite overwrap shows a smaller radial dimension together with axial extension. Consequently, a clear displacement gap develops between the two profiles, particularly over the domes and near the dome–cylinder transitions. The mismatch is also visible along the cylindrical section, where the liner tends to move inward smoothly, while the overwrap presents a circumferentially varying deformation. After the two components are tied, Figure 10b shows that the bonded vessel adopts an intermediate deformation mode. The cylindrical section still contracts radially, while the dome and polar region move outward in the axial direction. The maximum displacement decreases from approximately 13.00 mm in the independent state to 7.16 mm after bonding, indicating that neither component can maintain its preferred free thermal deformation.

A possible explanation is that the bonded response is governed by the mismatch between the nearly isotropic contraction of the liner and the anisotropic contraction of the composite overwrap, as schematically illustrated in Figure 11 [31]. During cooling, the liner tends to shorten axially and contract circumferentially, whereas the geodesic layers in the overwrap strongly restrain axial shortening and may promote slight axial extension as the vessel diameter decreases. After the two components are tied, these different free-deformation tendencies must become compatible [10]. The liner therefore suppresses the axial extension of the overwrap, while the overwrap restrains the free contraction of the liner, which may introduce additional tensile and bending stresses near the dome–cylinder transition [20]. This interpretation is consistent with Figure 10c, where a localized high-stress band appears near the shoulder region and the maximum principal stress reaches approximately 481.5 MPa. Along the cylindrical section, the hoop and geodesic layers provide different radial constraints because of their distinct fiber orientations and thermal contraction characteristics. The resulting nonuniform restraint may be transferred to the liner through the tied interface, producing the weak circumferential undulation observed in Figure 10b.

Figure 12 compares the cryogenic stress states of the vessel before and after the application of 1 MPa internal pressure. During free cooling, the liner exhibits a greater overall contraction than the composite overwrap in the deformation directions constrained by the bonded interface. Once tied, the liner is prevented from reaching its preferred contracted configuration and is therefore subjected to tensile thermal stress, whereas the overwrap experiences an opposing constraint that produces predominantly compressive fiber-direction stresses, together with local tensile stresses associated with the varying winding orientation and dome geometry. The subsequent application of internal pressure induces circumferential expansion and axial extension of the vessel. For the liner, subsequent pressurization modifies the pre-existing cooling-induced stress state, increasing the maximum von Mises stress from approximately 412.5 to 456.6 MPa under the combined thermal–pressure loading. In the composite overwrap, the tensile effect of internal pressure partly counteracts the cooling-induced compressive stress [11,32], reducing the extreme fiber-direction stresses from approximately +471.7/−438.6 MPa to +338.0/−370.1 MPa. The improved deformation compatibility between the two components is also accompanied by a reduction in the maximum von Mises stress of the innermost composite solid elements adjacent to the tied interface, from approximately 440.6 to 315.4 MPa, although localized stress concentrations remain near the boss and dome–cylinder transition regions. This quantity describes the local solid-material stress state near the bonded surface and is not used as an interface-failure measure.

### 3.3. Effect of Dome Aspect Ratio Under Cryogenic and Combined Loading

Having clarified the cryogenic response of the representative DAR = 1.25 vessel, the influence of dome geometry under the same cooling condition is further examined. Figure 13 shows an overall reduction in the extreme fiber-direction stress as DAR increases, although the individual tensile and compressive extrema exhibit local non-monotonic variations. The tensile maxima are approximately 471.7, 207.6, 88.3, 184.2, 152.5, and 150.3 MPa for DAR = 1.25, 1.50, 1.75, 2.00, 2.25, and 2.50, respectively. At smaller DAR values, the deeper dome produces a larger axial deformation difference between the dome and cylindrical sections, and this incompatibility is transferred through the continuously varying geodesic fibers, resulting in relatively high local fiber-direction stresses. As DAR increases, the dome height and meridional length decrease, so the thermal deformation mismatch accumulated between the polar region and the cylinder is generally reduced. However, the exact local extrema do not vary strictly monotonically because the local geodesic winding angle and thickness distribution also change with dome geometry [31]. The main effect of increasing DAR under pure cooling is therefore an overall reduction in the extreme fiber-direction stress level, accompanied by local fluctuations and a redistribution of the dome stress toward the dome–cylinder and boss transition regions.

Figure 14 presents a distinctly weaker dependence of the liner response on DAR. Both the von Mises stress level and its overall distribution remain broadly similar among the six configurations. The cylindrical section maintains an almost unchanged stress state, while variations in the dome are mainly reflected in the shape and position of the circumferential stress lobes rather than in a substantial change in the maximum value. This limited sensitivity arises because the liner material, thickness, cylindrical dimensions and temperature reduction are identical in all cases. Its cryogenic stress is governed primarily by the overall contraction mismatch with the composite overwrap, which changes only moderately with dome geometry. In addition, the isotropic liner redistributes the interfacial restraint in multiple directions, whereas the fiber-direction stress in the overwrap responds directly to changes in local winding orientation and deformation compatibility [12]. Consequently, DAR has a pronounced influence on the directional stress of the composite layer but only a limited effect on the equivalent stress of the metallic liner.

Building on the pure-cooling results, Figure 15 compares the fiber-direction stress distributions under 1 MPa internal pressure at room and cryogenic temperatures. At room temperature, the stress field is governed primarily by pressure loading, and increasing DAR gradually shifts the more pronounced stress regions from the dome surface toward the dome–cylinder transition. Under cryogenic conditions, the pre-existing thermal stress modifies this pressure-dominated pattern. For DAR = 1.25, extensive compressive regions remain over the dome and lower transition, whereas the corresponding room-temperature result is dominated by tensile fiber stress. As DAR increases, the longitudinal banded pattern remains evident over the cylindrical section and extends into the dome shoulder to different degrees, while the principal stress variation becomes increasingly associated with the dome–cylinder and boss transition regions. For DAR = 2.25 and 2.50, the stress distribution over the central cylindrical section appears comparatively stable, and the geometric influence is concentrated mainly near the two ends of the vessel. This difference from the room-temperature response results from the combined action of two competing effects. Internal pressure generates tensile fiber stress, whereas constrained cooling introduces predominantly compressive thermal stress into the overwrap. As established in Section 3.2, pressure partially offsets the cooling-induced compression, but the extent of this compensation varies with DAR because dome geometry changes the geodesic fiber path, deformation compatibility, and stress-transfer distance between the dome and cylindrical section.

Figure 16 further examines the circumferential stress in the outermost hoop layer. All six curves remain compressive and exhibit four nearly identical periodic cycles over the analyzed circumferential distance. The local maxima are approximately −10.4 to −11.0 MPa, whereas the minima are approximately −92.3 to −92.9 MPa, giving a fluctuation range of about 82 MPa. The differences among the six DAR values are below approximately 0.7 MPa at both the peaks and troughs, and the curves almost completely overlap over the full path. This result confirms that the circumferential fluctuation observed in the cylindrical section is controlled primarily by the local winding architecture and the alternating restraint transferred through the bonded interface. Because the cylindrical diameter, hoop-layer geometry and stacking sequence are identical in all models, changing the dome shape has little influence on the stress state of the outer hoop layer sufficiently far from the transition region.

A more detailed dependence on DAR is revealed by the fiber-direction stress along the centered axial profile, as shown in Figure 17. Because the cylindrical sections are aligned and have identical dimensions and layups, the six curves can be compared directly within the same structural regions. Over the central part of the cylinder, they converge to a relatively stable compressive level of approximately −190 to −200 MPa, indicating that DAR has only a limited influence away from the two domes. The curves begin to separate near the dome–cylinder transitions. At the right transition, the stress rises progressively from approximately −120 MPa for DAR = 1.25 to slight tension for DAR = 2.50. This systematic shift suggests that the balance between cooling-induced compression and pressure-induced tension changes with dome geometry. As DAR increases and the dome becomes shallower, the compressive thermal contribution near the transition is reduced, while the pressure-induced tensile response becomes more pronounced.

Within the dome region, the stress varies over a much wider range, from slight tension to below −300 MPa in compression, and exhibits repeated tensile–compressive reversals. Smaller-DAR vessels generally show broader highly compressed regions, with DAR = 1.25 reaching approximately −320 MPa in the left dome and about −290 MPa in the right dome. As DAR increases, the dome length decreases and the stress variation is confined to a shorter meridional distance, resulting in steeper gradients and more localized fluctuations near the boss and dome–cylinder junctions. The local extrema do not change strictly monotonically, probably because they are also affected by the continuously varying geodesic angle and local thickness distribution [31]. These results show that DAR has little effect on the established stress level in the cylinder core, but substantially changes the magnitude, sign and spatial concentration of the fiber-direction stress in the transition and dome regions under coupled cryogenic and pressure loading.

Within the present modelling framework, the results are intended primarily for comparing DAR-dependent thermomechanical responses rather than for predicting progressive damage or ultimate structural capacity. Temperature-dependent composite failure, local liner plasticity, and interfacial debonding are not explicitly modelled; accordingly, the reported stresses and displacements are interpreted mainly as comparative indicators of stress redistribution, deformation compatibility, and geometry-sensitive regions. To provide a direct quantitative comparison across the complete DAR range within this scope, the principal response quantities under the three loading conditions are summarized in Table 3.

The quantitative comparison in Table 3 further demonstrates that the influence of DAR depends strongly on both loading condition and response quantity. Under room-temperature pressurization, the maximum liner stress remains nearly unchanged at approximately 182.5 MPa for DAR = 1.25–1.75 but subsequently increases to 366.0 MPa at DAR = 2.50, accompanied by an increase in maximum displacement from 1.330 to 7.250 mm. Under pure cooling, the liner maximum varies only modestly, whereas the fiber-direction stress is substantially more sensitive to DAR and exhibits an overall reduction accompanied by local non-monotonic fluctuations; the maximum displacement decreases continuously from 7.125 to 4.981 mm. Under cryogenic pressurization, the liner maximum gradually increases with DAR, while the minimum tensile fiber-direction stress and minimum displacement both occur around DAR = 2.00. These results indicate that no single DAR minimizes all response measures and further emphasize that dome geometry should be evaluated according to the governing thermomechanical condition and multiple structural-response indicators.

## 4. Conclusions

This study numerically investigated the thermomechanical response of a large-scale metal-lined composite overwrapped pressure vessel with six ellipsoidal dome aspect ratios (DARs) under pressurization at 293 K, cryogenic cooling from 293 to 90 K, and combined cryogenic pressurization. The main conclusions are as follows.

(1) Under 1 MPa internal pressure at 293 K, increasing DAR makes the dome shallower and shortens the load-transfer region between the dome and cylindrical section. As a result, the stress distribution becomes increasingly localized near the dome–cylinder transitions. The maximum tensile fiber-direction stress in the composite overwrap increases from approximately 73.4 MPa at DAR = 1.25 to 299.2 MPa at DAR = 2.50, while the maximum von Mises stress in the liner reaches approximately 355.7 MPa at DAR = 2.50. This localization becomes particularly evident when DAR exceeds approximately 2.00.
(2) Cryogenic cooling produces fundamentally different free-deformation tendencies in the metallic liner and composite overwrap. The nearly isotropic liner contracts in both the axial and circumferential directions, whereas the anisotropic overwrap contracts circumferentially but tends to extend axially in the cylindrical section because of the low fiber-direction thermal expansion and the geometric response of the helical winding paths. After bonding, this deformation mismatch is constrained, generating substantial thermal stresses near the dome–cylinder and boss transition regions.
(3) The application of internal pressure after cooling affects the two components differently. In the liner, subsequent pressurization acts together with the pre-existing cooling-induced stress state, increasing the maximum von Mises stress from approximately 412.5 to 456.6 MPa. In the composite overwrap, the tensile stress generated by internal pressure partly offsets the cooling-induced compressive stress, reducing the extreme fiber-direction stresses from approximately +471.7/−438.6 MPa to +338.0/−370.1 MPa. Internal pressure therefore intensifies the liner stress while partially alleviating the extreme thermal stress in the overwrap.
(4) Under cryogenic and combined loading, the influence of DAR is concentrated mainly in the domes and transition regions, whereas the central cylindrical section remains comparatively insensitive to dome geometry. Increasing DAR generally reduces the extreme fiber-direction stress induced by cooling, but also produces steeper stress gradients and stronger localization near the dome–cylinder transitions under pressurization. Therefore, DAR should be selected by jointly considering thermal mismatch, pressure-induced stress localization and deformation compatibility, rather than solely according to room-temperature pressure performance.
(5) The preferred DAR tendency depends on the governing loading condition. Lower DAR values reduce pressure-induced stress localization near the dome–cylinder transition, whereas higher DAR values generally reduce the extreme composite thermal stress during cryogenic cooling. Under combined loading, the response is less monotonic, indicating that DAR selection should consider multiple loading conditions and additional design objectives rather than a single stress measure.

## Figures and Tables

**Figure 1:**
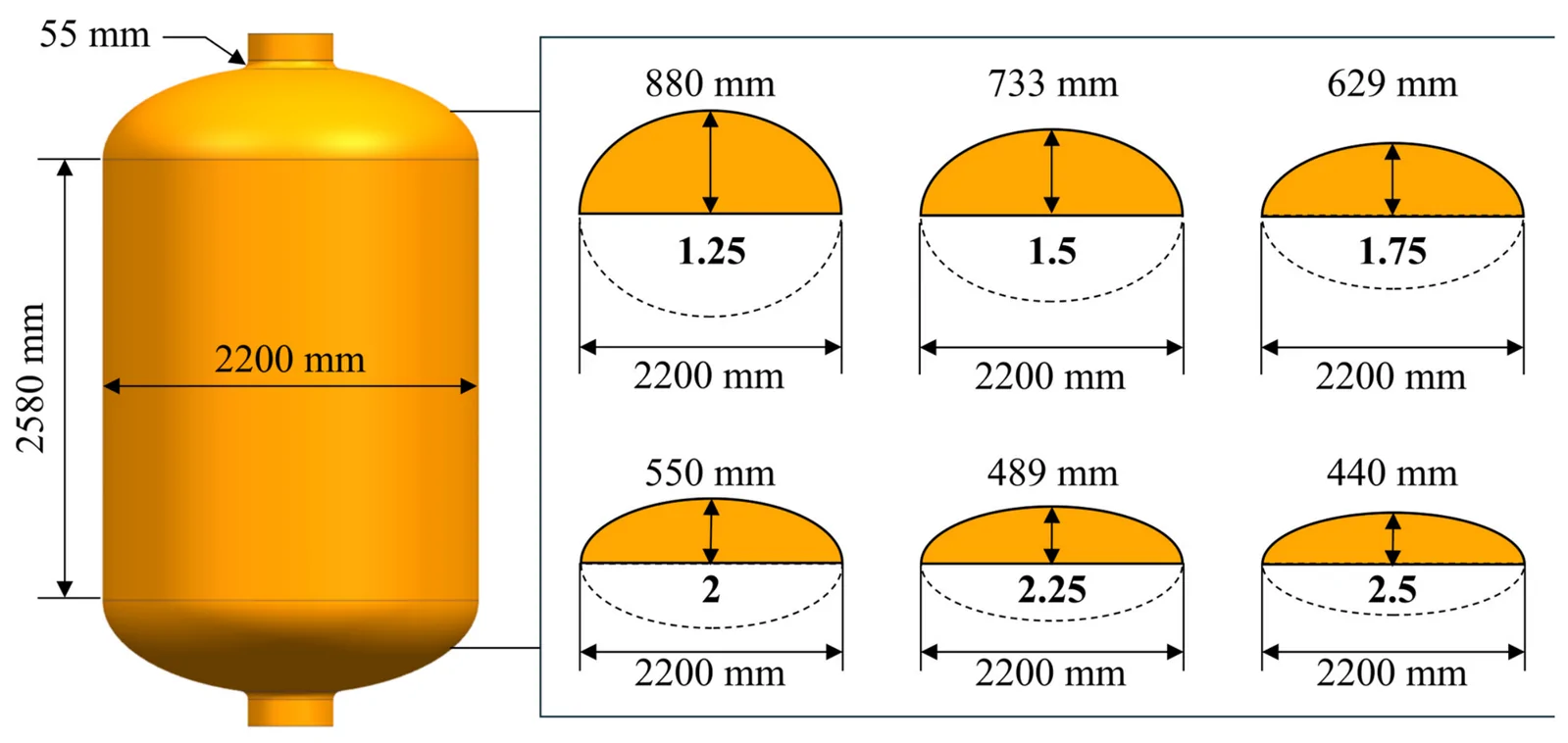
Geometrical configuration of the vessel and definition of the dome aspect ratio (DAR).

**Figure 2:**
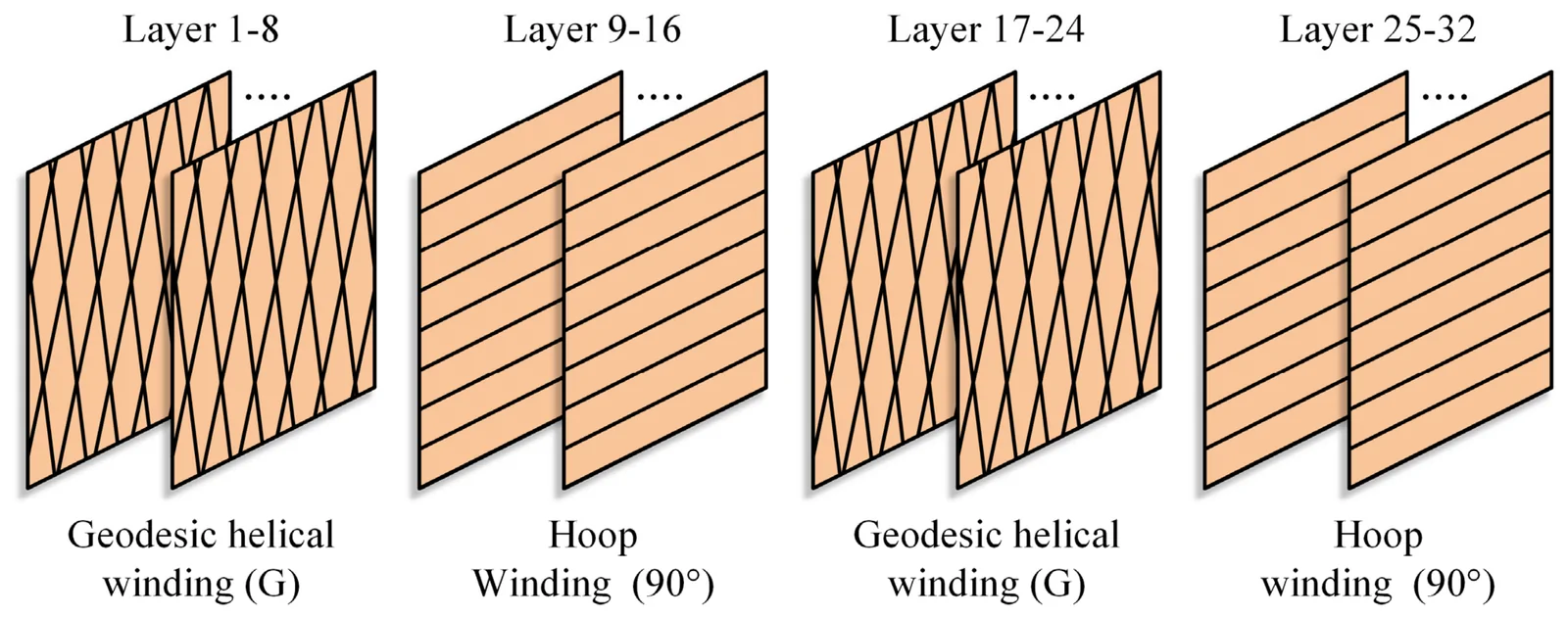
Composite winding sequence and equivalent-layer representation.

**Figure 3:**
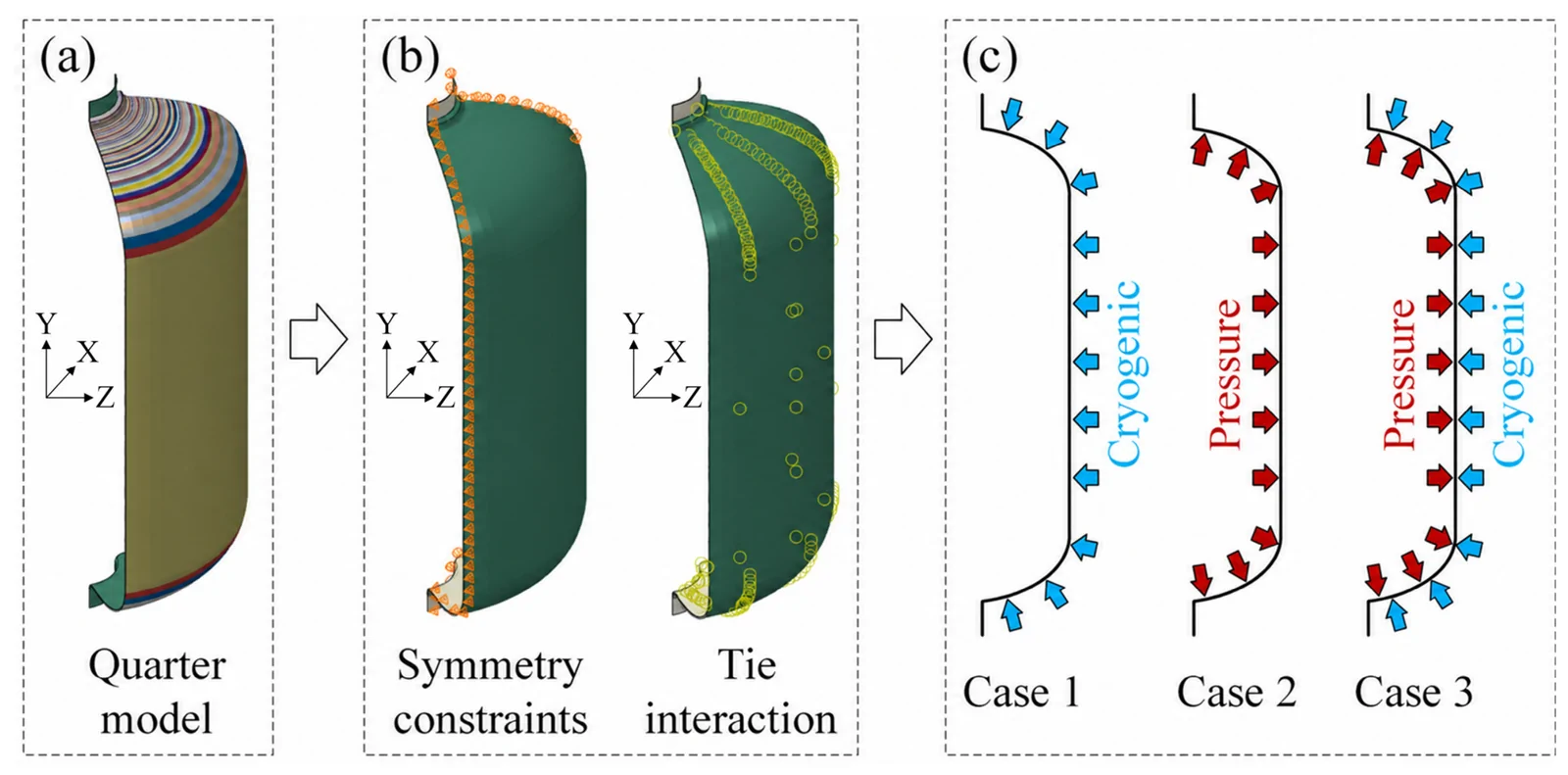
Finite element model and analysis settings: (**a**) quarter model and mesh; (**b**) symmetry constraints and tie interaction; (**c**) cryogenic, pressure, and combined loading cases.

**Figure 4:**
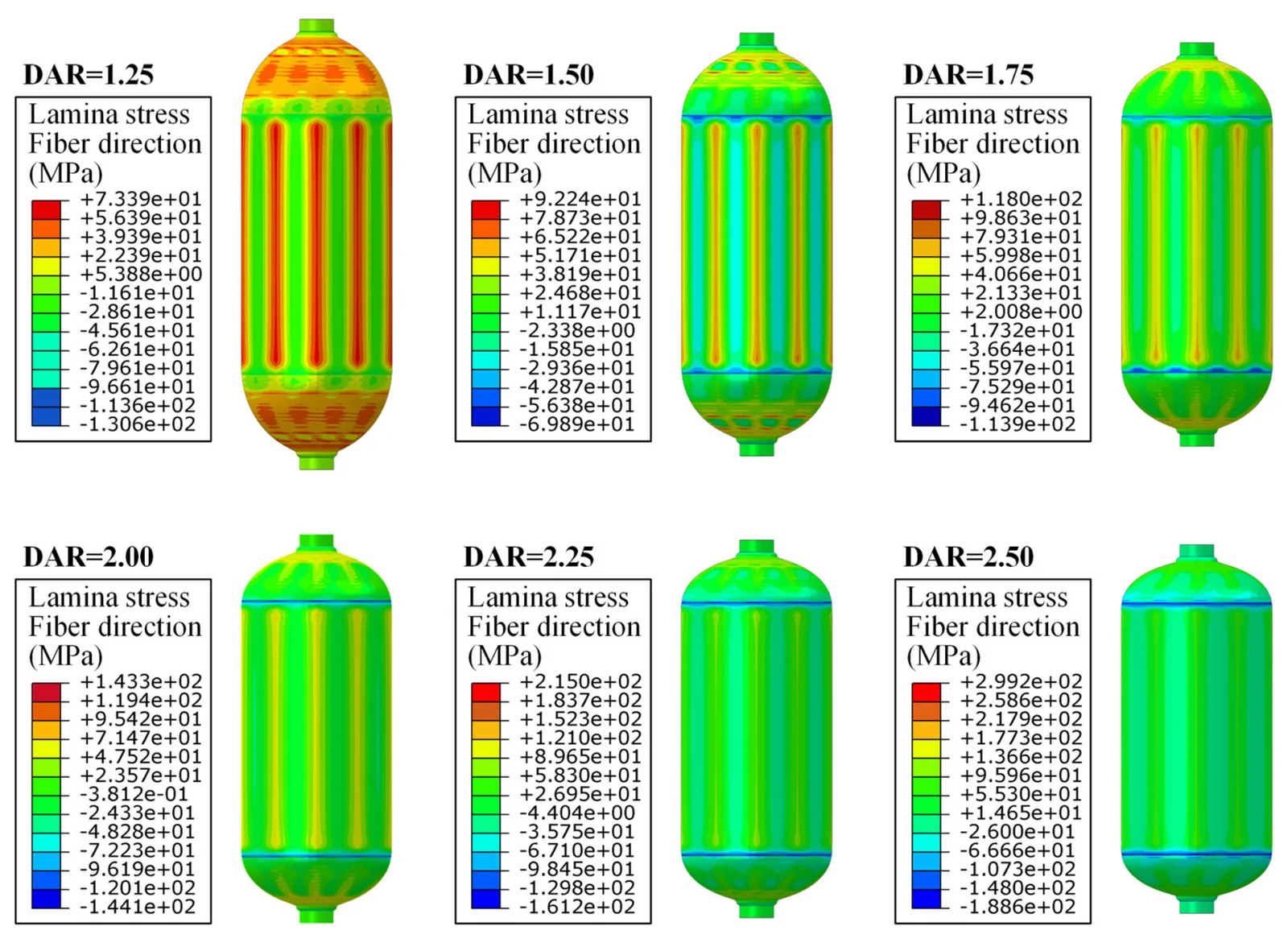
Fiber-direction stress distributions in the composite overwrap for different DAR values under room-temperature pressurization at 1 MPa.

**Figure 5:**
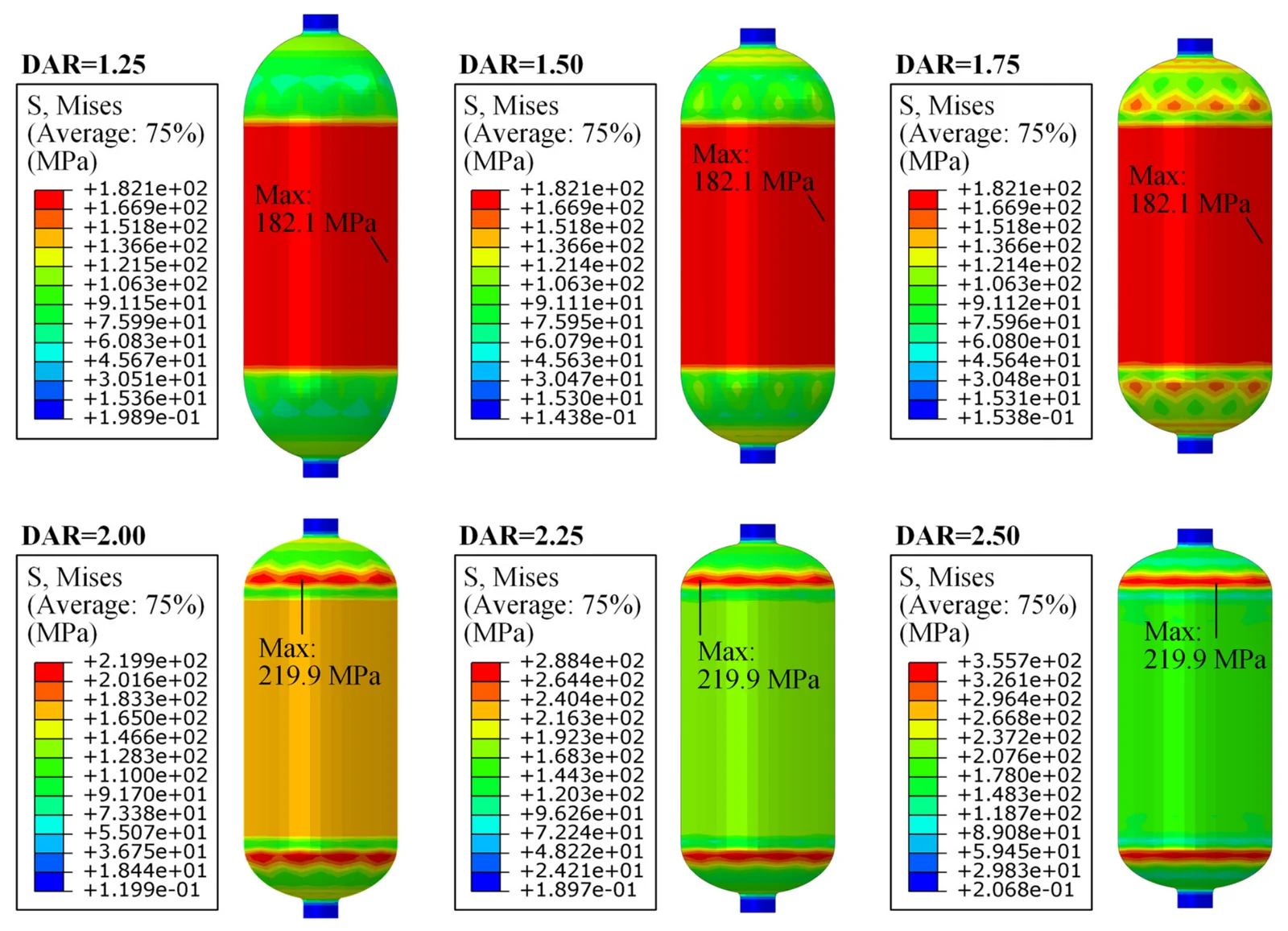
Von Mises stress distributions in the metallic liner for different DAR values under room-temperature pressurization at 1 MPa.

**Figure 6:**
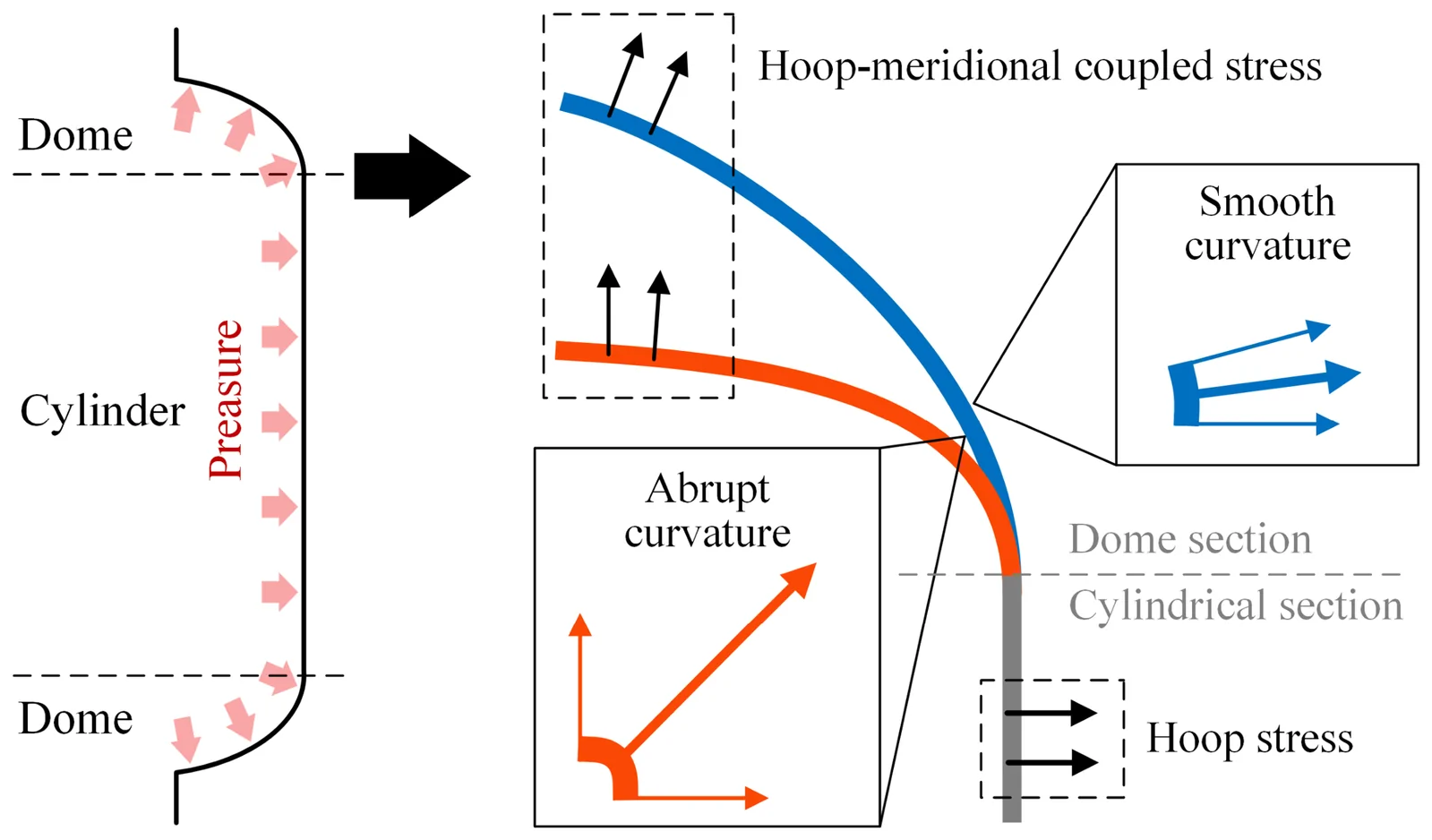
Schematic illustration of the coupled geometric and winding-related stress concentration at the dome–cylinder junction.

**Figure 7:**
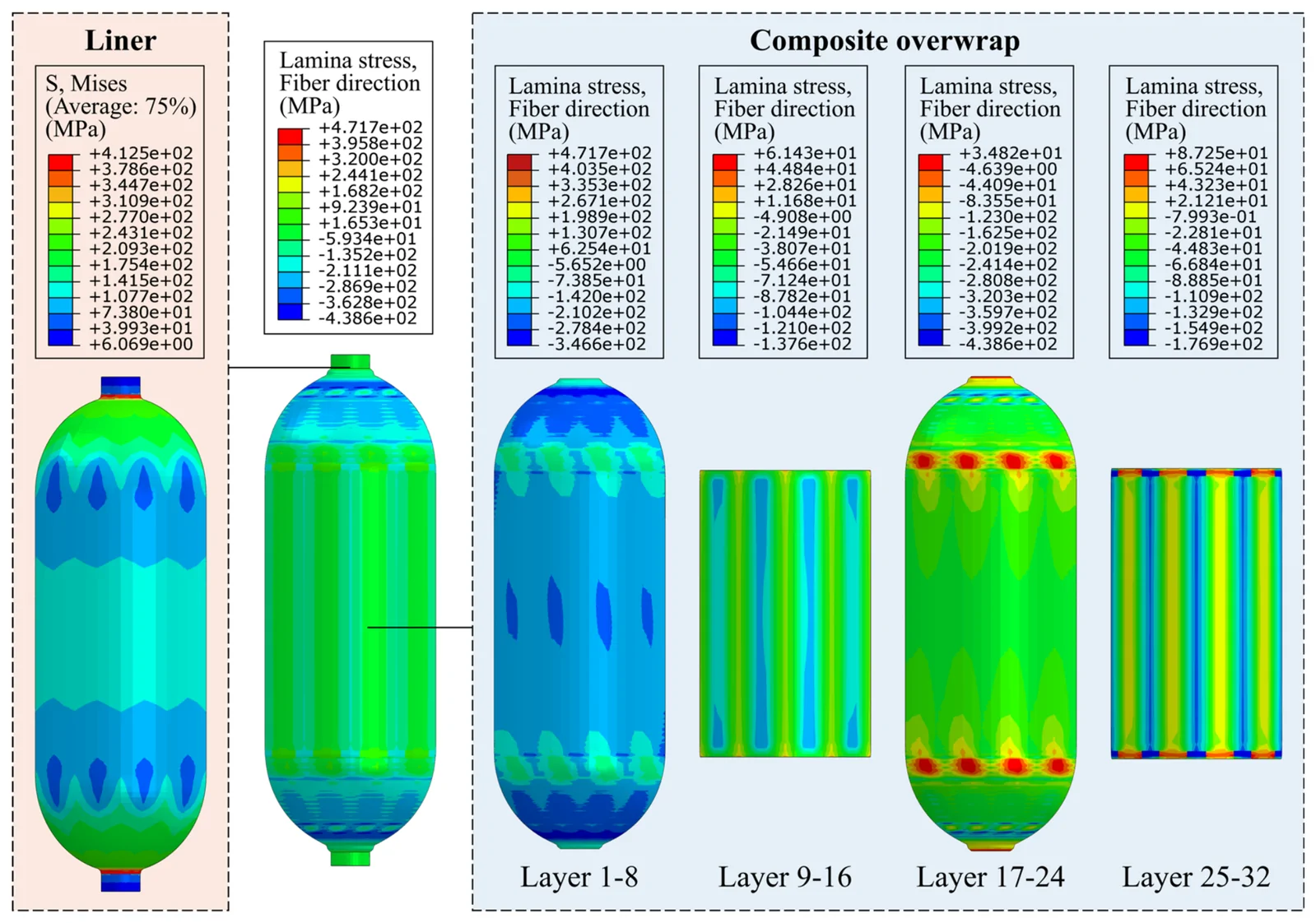
Cryogenic stress distributions in the liner and composite overwrap at DAR = 1.25.

**Figure 8:**
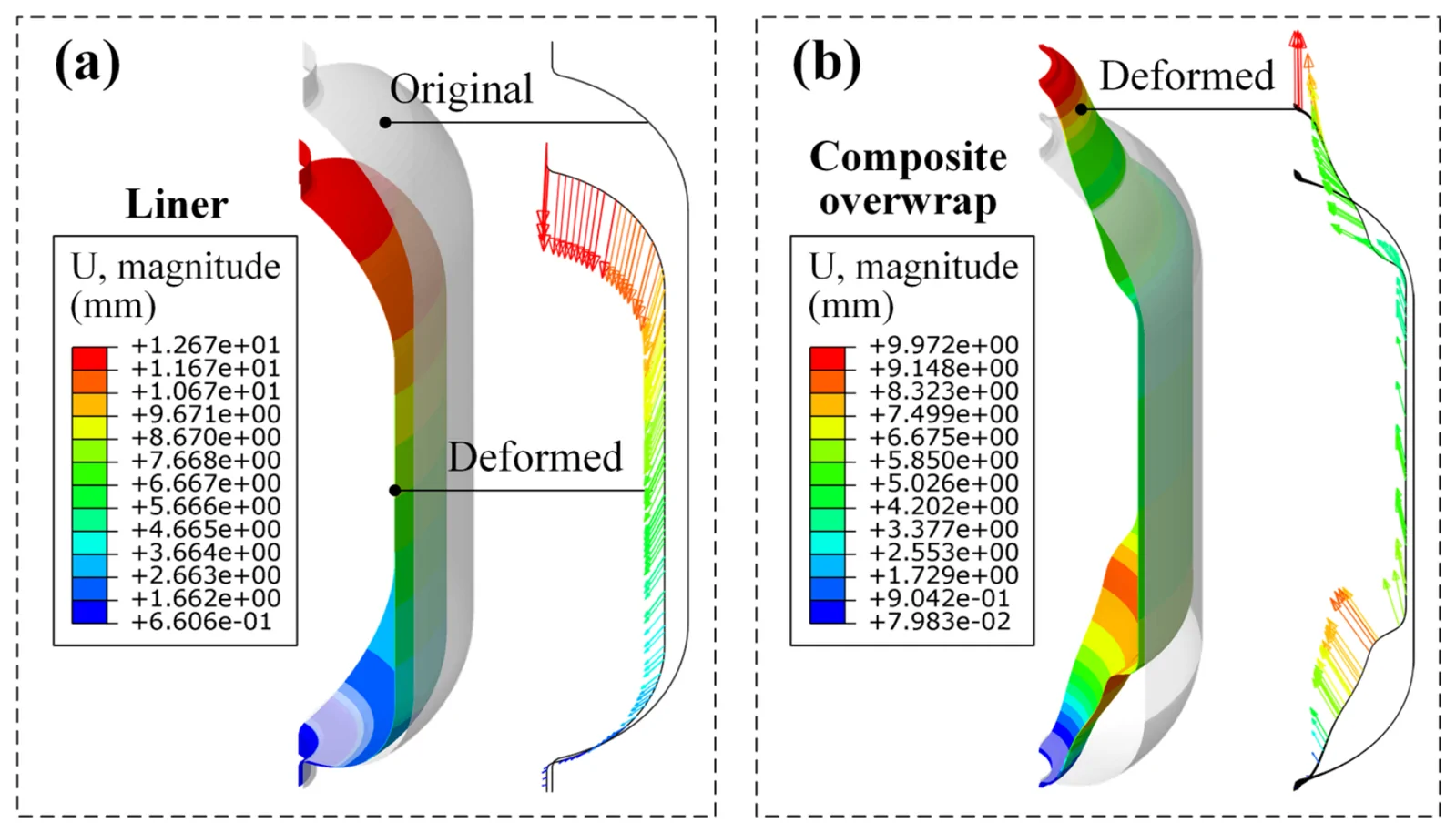
Free thermal deformation magnified 50 times for the liner and composite overwrap at DAR = 1.25, including (**a**) the liner and (**b**) the composite overwrap.

**Figure 9:**
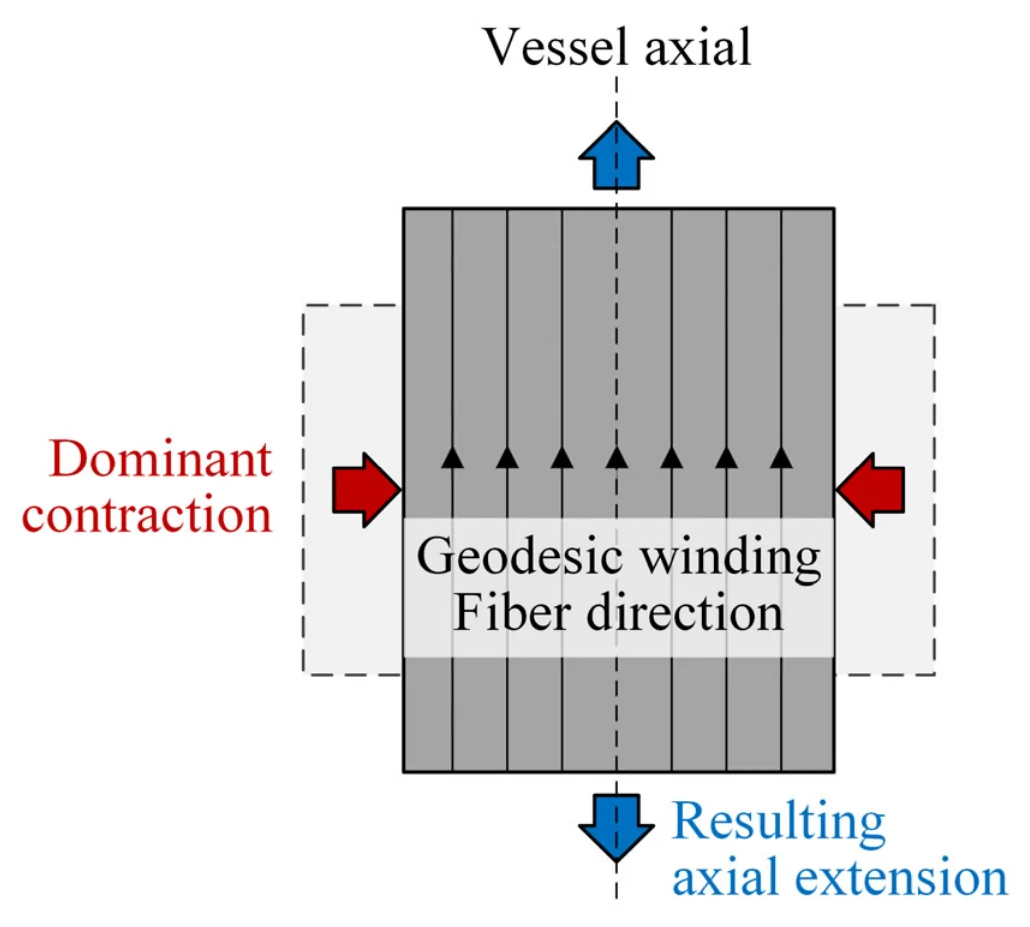
Schematic illustration of axial extension induced by circumferential contraction in the composite overwrap.

**Figure 10:**
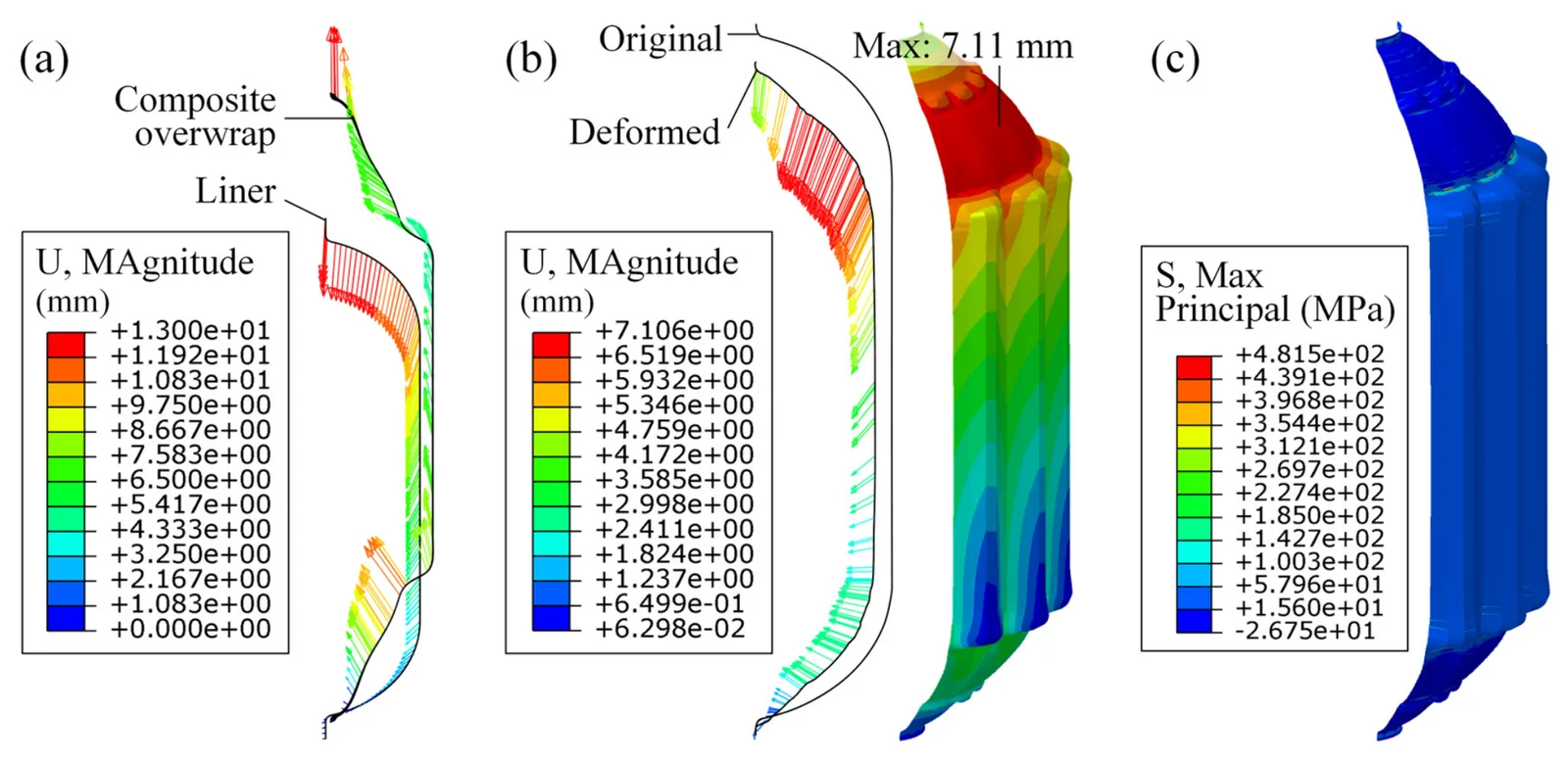
Free and bonded thermal responses magnified 50 times at DAR = 1.25, including (**a**) overlaid free-deformation profiles, (**b**) bonded deformation, and (**c**) maximum principal stress after bonding.

**Figure 11:**
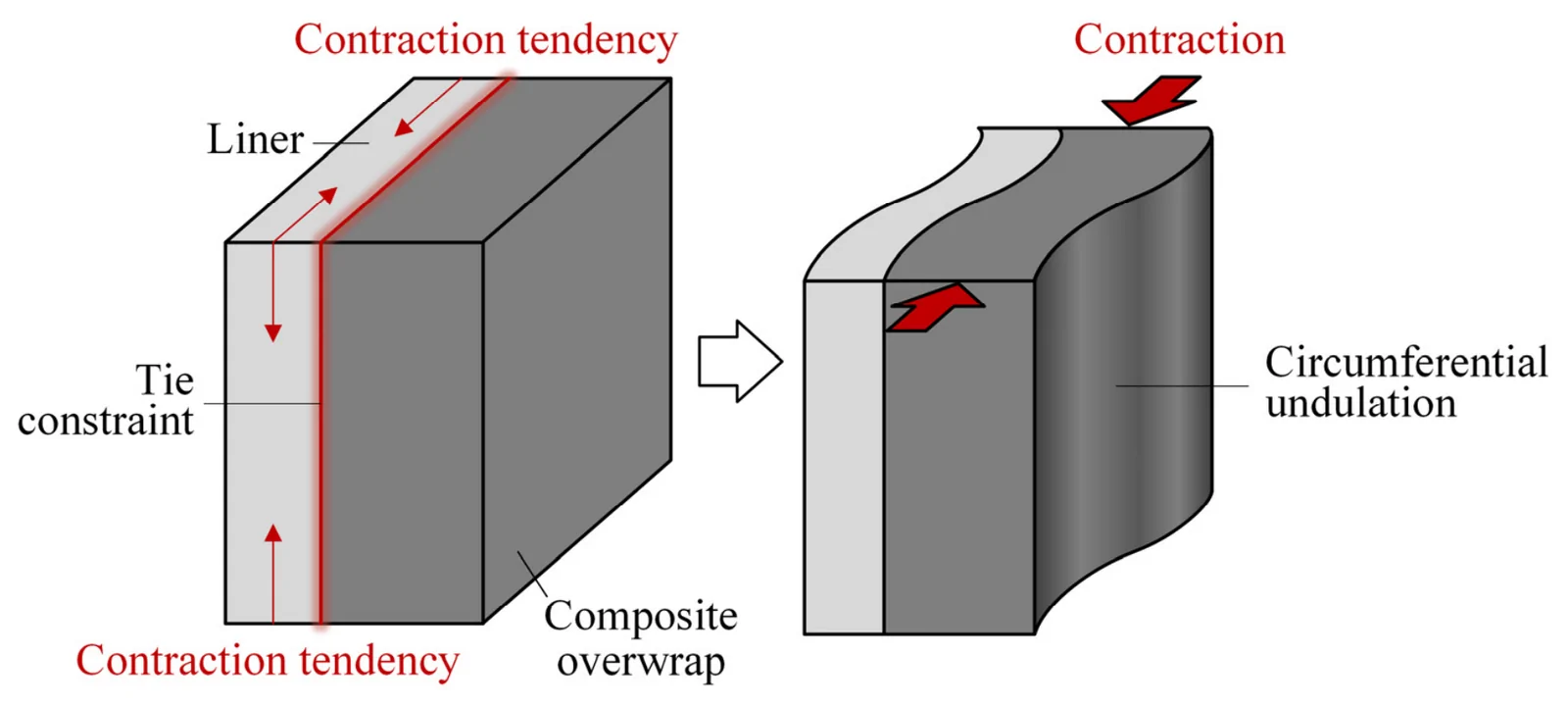
Schematic illustration of thermally induced contraction mismatch and circumferential undulation in the bonded liner–COPV system.

**Figure 12:**
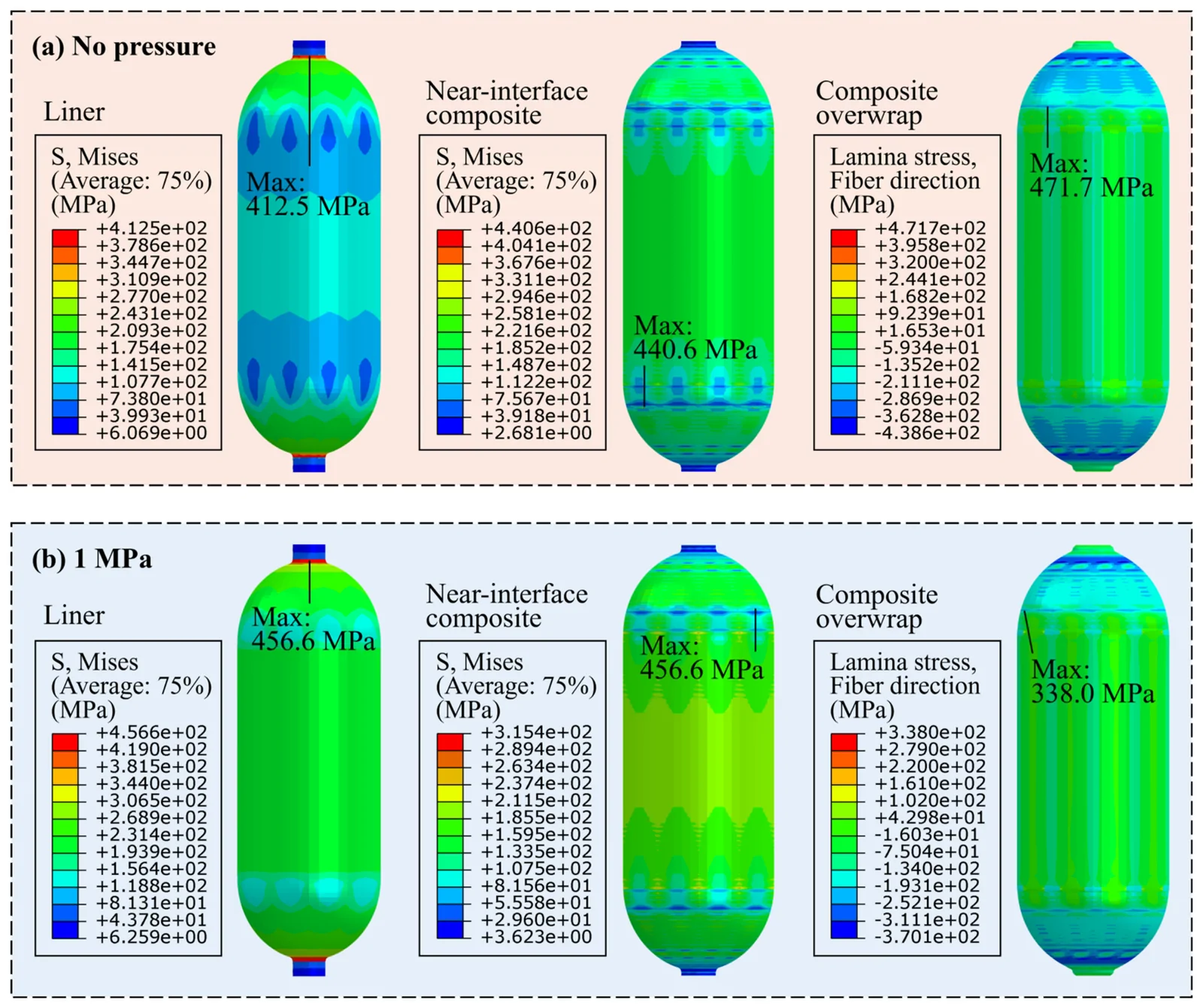
Cryogenic stress distributions in the liner, the innermost composite solid elements adjacent to the tied interface, and the composite overwrap at DAR = 1.25 under (**a**) no pressure and (**b**) 1 MPa internal pressure.

**Figure 13:**
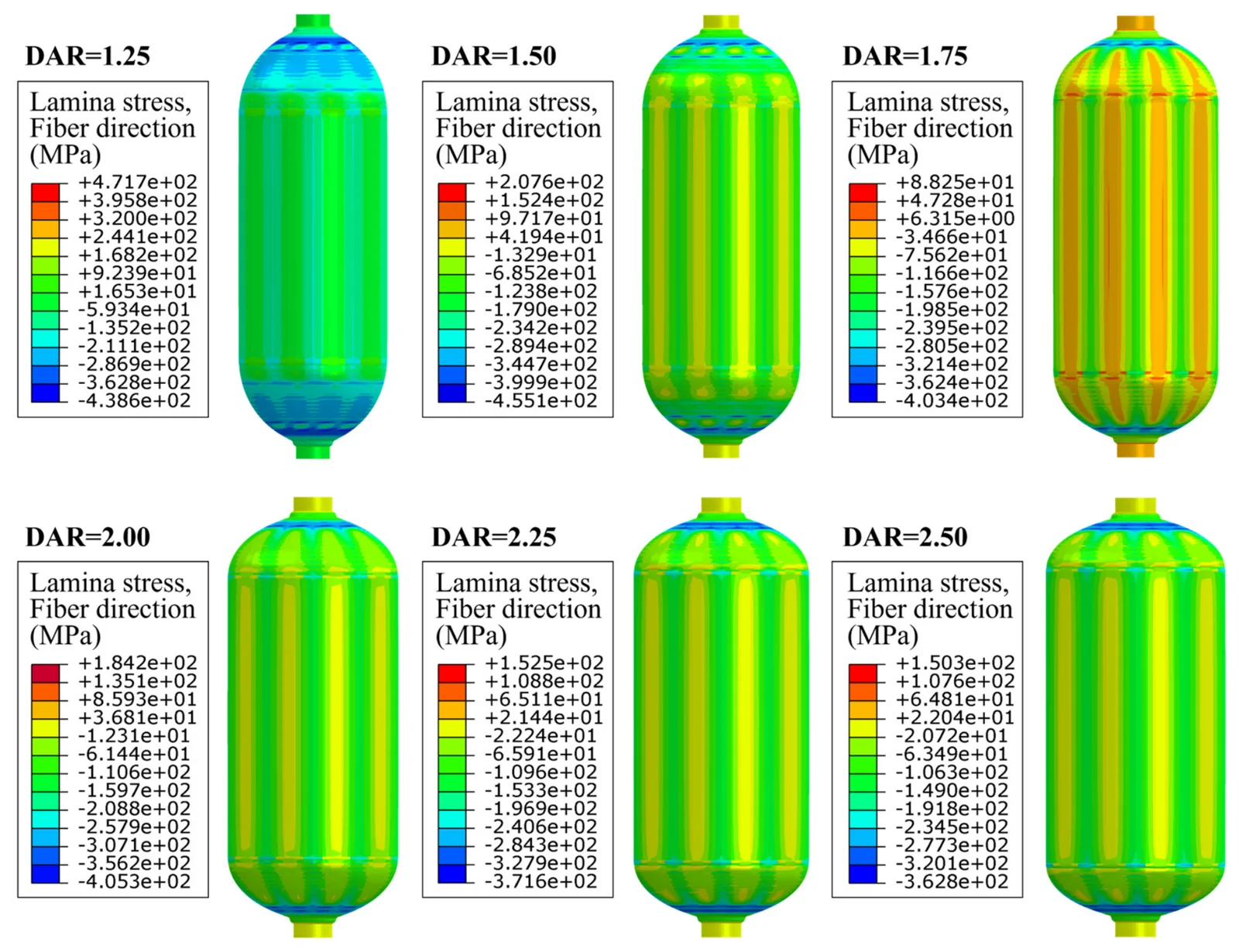
Fiber-direction stress distributions in the composite overwrap for different DAR values under cryogenic cooling without internal pressure.

**Figure 14:**
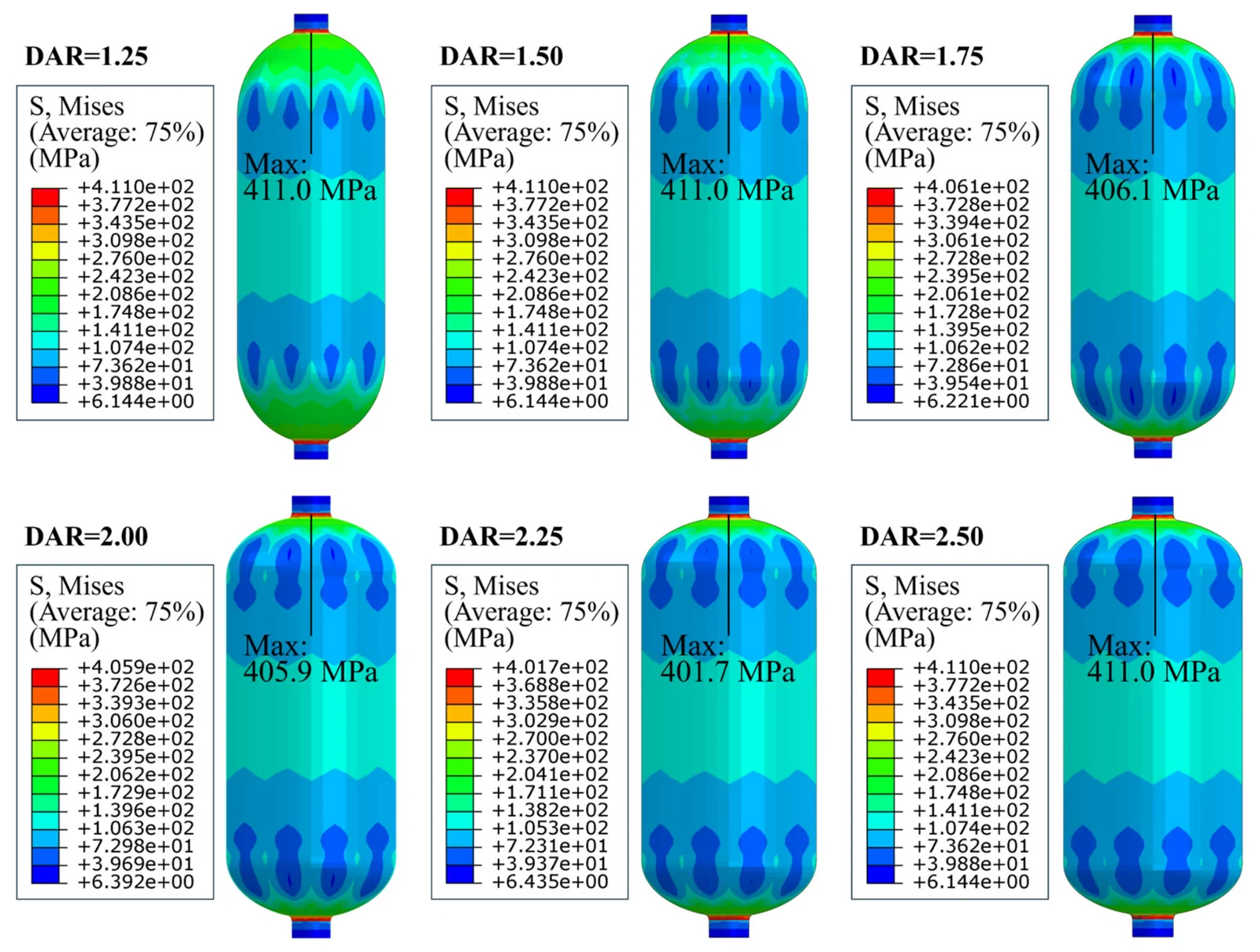
Von Mises stress distributions in the metallic liner for different DAR values under cryogenic cooling without internal pressure.

**Figure 15:**
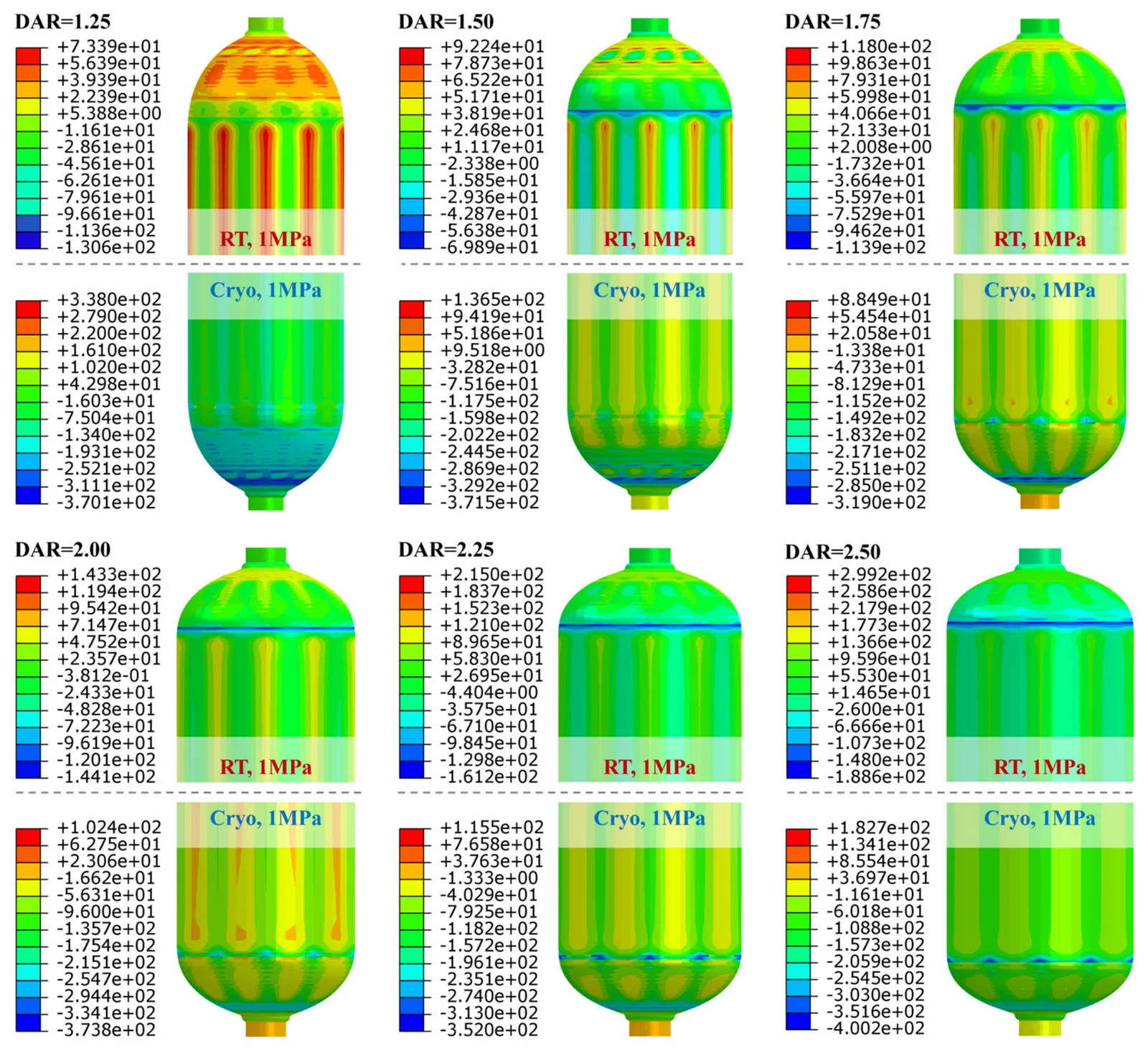
Fiber-direction stress distributions for different DAR values under room-temperature and cryogenic pressurization at 1 MPa; stress values are given in MPa.

**Figure 16:**
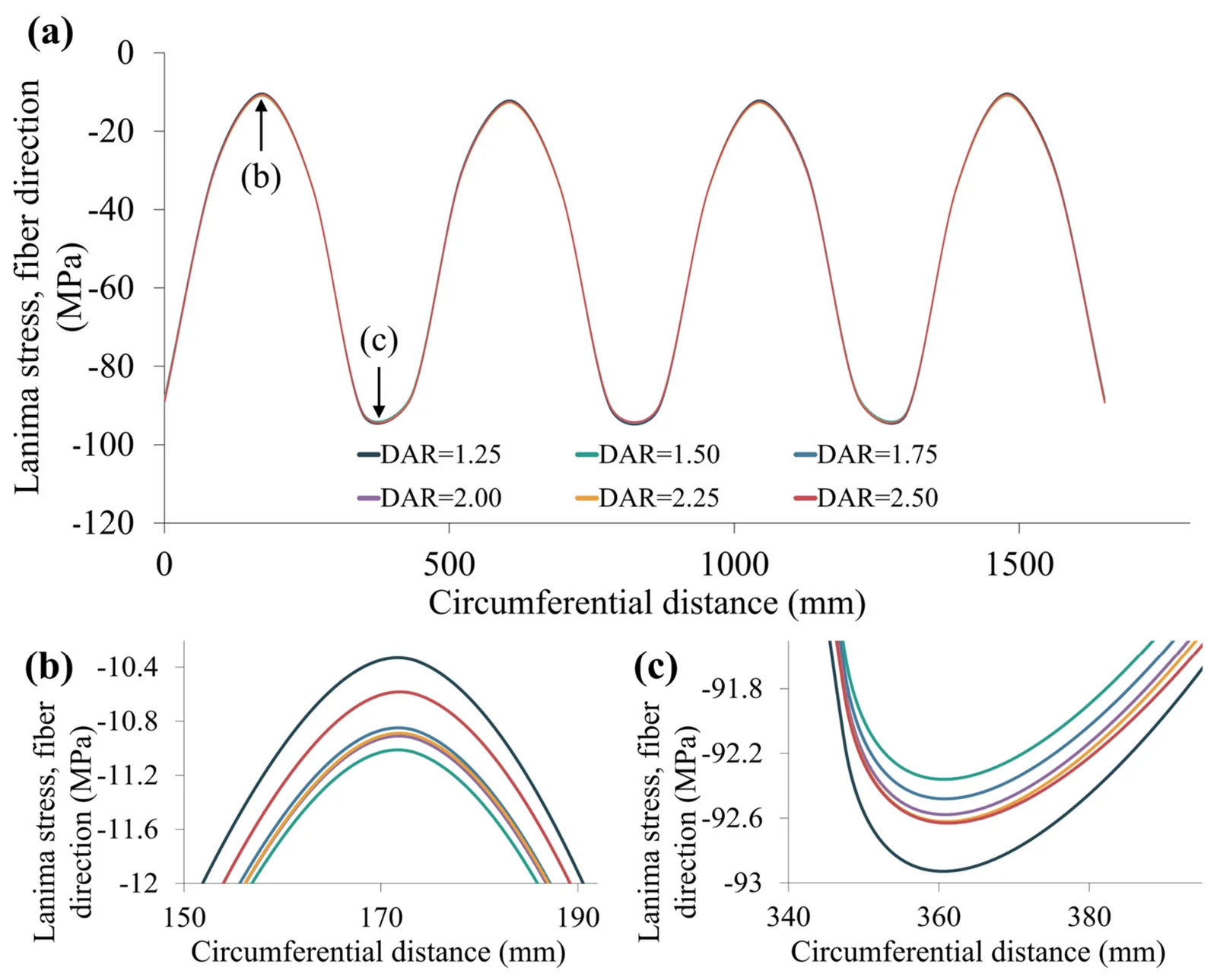
Circumferential fiber-direction stress in the outermost hoop layer for different DAR values under cryogenic pressurization at 1 MPa, including (**a**) the complete circumferential profile and (**b**,**c**) enlarged local variations.

**Figure 17:**
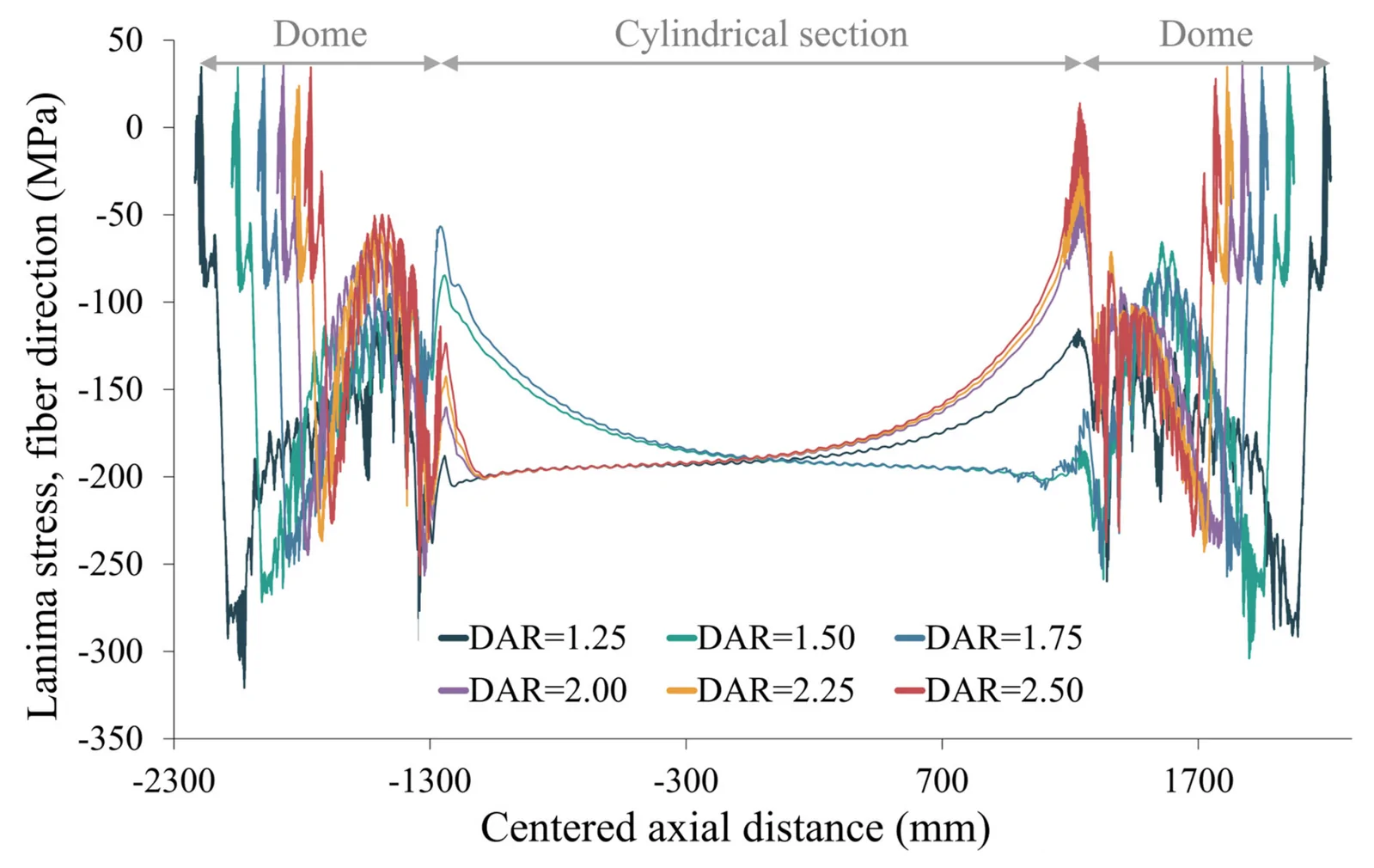
Fiber-direction stress along the centered outer axial profile for different DAR values under cryogenic pressurization at 1 MPa.

**Table 1:** Material properties of the 304 stainless steel liner [25].

Property	293 K	193 K	90 K
Density (tonne/mm^3^)	7.90 × 10^−9^	7.90 × 10^−9^	7.90 × 10^−9^
Young’s modulus (MPa)	199,500	207,700	214,100
Poisson’s ratio	0.27	0.27	0.27
Thermal expansion coefficient (K^−1^)	1.33 × 10^−5^	1.33 × 10^−5^	1.33 × 10^−5^

**Table 2:** Temperature-dependent properties of the T700-grade carbon/epoxy composite [12,26,27], with direction 1 along the fiber and directions 2 and 3 transverse to the fiber.

Property	293 K	193 K	90 K
Density (tonne/mm^3^)	1.80 × 10^−9^	1.80 × 10^−9^	1.80 × 10^−9^
E_1_ (MPa)	147,700	147,700	147,700
E_2_ (MPa)	8520	10,050	12,350
E_3_ (MPa)	8520	10,050	12,350
ν_12_	0.30	0.30	0.30
ν_13_	0.30	0.30	0.30
ν_23_	0.30	0.30	0.30
G_12_ (MPa)	4590	6020	8630
G_13_ (MPa)	4590	6020	8630
G_23_ (MPa)	4590	6020	8630
α_11_ (K^−1^)	3.1 × 10^−6^	3.1 × 10^−6^	3.1 × 10^−6^
α_22_ (K^−1^)	24.7 × 10^−6^	24.7 × 10^−6^	24.7 × 10^−6^
α_33_ (K^−1^)	24.7 × 10^−6^	24.7 × 10^−6^	24.7 × 10^−6^

**Table 3:** Quantitative summary of the principal thermomechanical responses for different DAR values and loading conditions.

Loading Condition	DAR	Max. Liner Von Mises Stress (MPa)	Fiber-Direction Stress Max./Min. (MPa)	Max. Displacement (mm)
293 K 1 MPa	1.25	182.5	+67.7/−123.9	1.330
	1.50	182.5	+89.5/−85.0	1.519
	1.75	182.5	+103.3/−75.1	2.688
	2.00	227.6	+111.8/−92.0	3.959
	2.25	290.3	+178.0/−94.8	5.445
	2.50	366.0	+244.1/−116.8	7.250
90 K, no pressure	1.25	412.5	+475.0/−311.1	7.125
	1.50	411.0	+306.3/−359.1	6.937
	1.75	406.1	+146.9/−255.7	6.539
	2.00	405.9	+191.1/−251.4	5.963
	2.25	401.7	+162.6/−256.6	5.412
	2.50	400.5	+171.6/−252.2	4.981
90 K 1 MPa	1.25	456.6	+344.1/−252.9	6.640
	1.50	462.6	+210.8/−267.9	6.114
	1.75	465.1	+114.3/−210.1	5.677
	2.00	471.8	+112.3/−275.5	5.245
	2.25	477.1	+161.9/−264.1	6.224
	2.50	489.9	+169.1/−296.9	7.326

## Data Availability

The raw data supporting the conclusions of this article will be made available by the authors on request.
